# Validation of the 4AT, a new instrument for rapid delirium screening: a study in 234 hospitalised older people

**DOI:** 10.1093/ageing/afu021

**Published:** 2014-03-02

**Authors:** Giuseppe Bellelli, Alessandro Morandi, Daniel H.J. Davis, Paolo Mazzola, Renato Turco, Simona Gentile, Tracy Ryan, Helen Cash, Fabio Guerini, Tiziana Torpilliesi, Francesco Del Santo, Marco Trabucchi, Giorgio Annoni, Alasdair M.J. MacLullich

**Affiliations:** 1 Department of Health Sciences, University of Milano-Bicocca and S. Gerardo Hospital, Geriatric Clinic, Monza, Italy; 2 Geriatric Research Group, via Romanino 1, Brescia, Italy; 3 Department of Rehabilitation and Aged Care, Ancelle Della Carità Hospital, Cremona, Italy; 4 Department of Public Health and Primary Care, University of Cambridge, Cambridge, UK; 5 Centre for Cognitive Ageing and Cognitive Epidemiology, University of Edinburgh, Edinburgh, UK; 6 Edinburgh Delirium Research Group, Geriatric Medicine Unit, University of Edinburgh, Edinburgh, UK; 7 Department of Medicine, University of Roma Tor Vergata, Roma, Italy

**Keywords:** delirium, cognitive impairment, screening, geriatrics, dementia, older people, validation, delirium detection, diagnostic accuracy

## Abstract

**Objective:** to evaluate the performance of the 4 ‘A’s Test (4AT) in screening for delirium in older patients. The 4AT is a new test for rapid screening of delirium in routine clinical practice.

**Design**: prospective study of consecutively admitted elderly patients with independent 4AT and reference standard assessments.

**Setting**: an acute geriatrics ward and a department of rehabilitation.

**Participants:** two hundred and thirty-six patients (aged ≥70 years) consecutively admitted over a period of 4 months.

**Measurements:** in each centre, the 4AT was administered by a geriatrician to eligible patients within 24 h of admission. Reference standard delirium diagnosis (DSM-IV-TR criteria) was obtained within 30 min by a different geriatrician who was blind to the 4AT score. The presence of dementia was assessed using the Alzheimer's Questionnaire and the informant section of the Clinical Dementia Rating scale. The main outcome measure was the accuracy of the 4AT in diagnosing delirium.

**Results:** patients were 83.9 ± 6.1 years old, and the majority were women (64%). Delirium was detected in 12.3% (*n* = 29), dementia in 31.2% (*n* = 74) and a combination of both in 7.2% (*n* = 17). The 4AT had a sensitivity of 89.7% and specificity 84.1% for delirium. The areas under the receiver operating characteristic curves for delirium diagnosis were 0.93 in the whole population, 0.92 in patients without dementia and 0.89 in patients with dementia.

**Conclusions:** the 4AT is a sensitive and specific method of screening for delirium in hospitalised older people. Its brevity and simplicity support its use in routine clinical practice.

## Introduction

Delirium is a serious neuropsychiatric syndrome characterised by acute and fluctuating inattention, other cognitive deficits and alterations in level of consciousness [1]. It affects 11–30% of hospitalised older patients [2]. Delirium is independently associated with several adverse outcomes, including elevated costs, increased length of stay, long-term cognitive and functional decline, increased risk of institutionalisation, higher mortality, and patient and carer distress [3–7]. Recognition of delirium can improve outcomes [8]. For these reasons, detection is important. Yet 50–75% of delirium is undetected or misdiagnosed in acute hospitals [6–9].

The 4 ‘A’s Test (4AT; www.the4AT.com) is a new screening tool for delirium. It also incorporates two simple cognitive screening items. It was developed because, though many reliable and valid delirium screening tools exist, none appeared to have all the following features important in routine, non-specialist care: brevity (generally <2 min), no special training required, simple to administer (including in people with visual or hearing impairment), does not require physical responses, allows for assessment of ‘untestable’ patients (those who cannot undergo cognitive testing or interview because of severe drowsiness or agitation) and incorporates general cognitive screening to avoid the need for separate tools for delirium and other causes of cognitive impairment. The 4AT underwent several waves of piloting, and is already in use in multiple hospitals in the UK and internationally. Here we aimed to test the diagnostic accuracy of the 4AT against a reference standard in two populations of older hospitalised patients.

## Methods

This study adheres to the Standards for Reporting Diagnostic Accuracy statement (www.stard-statement.org). It was a cross-sectional observational study carried out at (i) the Geriatric Medicine Unit at the University of Milano-Bicocca and S. Gerardo Hospital, Monza and (ii) the Department of Rehabilitation and Aged Care (DRAC), Ancelle della Carità Hospital, Cremona, Italy, from 1 May to 31 August 2012. The Geriatric Medicine Unit is a 38-bedded acute geriatric medicine ward (total no. of patients/year 2012 = 1209) in a university hospital, admitting patients mostly from the Emergency Department. It also incorporates a six-bedded Orthogeriatrics Unit (OGU) for elderly patients with hip fracture [10]. The DRAC is an 80-bedded ward for inpatient rehabilitation of post-acute and chronic disability in older patients [4]. The most frequent reasons for admission are post-surgical care, stroke, chronic heart failure and pulmonary diseases, Parkinson's disease, or gait/balance disorders.

All consecutively admitted patients aged 70 years or above were eligible for inclusion. Exclusion criteria were patients with no verbal communication, or comatose [according to a score of −4 or less at the Richmond Agitation and Sedation Scale (RASS)] [11], learning disability, severe hearing disability, unable to speak Italian and lack of interpreter or no informed consent. The institutional review board of the Ethics Committee of the University of Milano-Bicocca approved the study. Informed consent from patients or their legal proxies was obtained.

### The 4AT

The 4AT comprises four items. Item 1 assesses level of alertness [12]. The next two items are brief cognitive screening tests: the Abbreviated Mental Test—4 (AMT4) [13], and attention testing with Months Backwards [14]. Item 4 assesses acute change or fluctuation in mental status [1]. The 4AT is scored from 0 to 12. A score of 0 is intended to suggest that delirium and/or moderate to severe cognitive impairment is unlikely, though the latter possibility is not being evaluated in the present study. Scores between 1 and 3 are intended to suggest possible moderate to severe general cognitive impairment (that is, corresponding to moderate to severe impairment on standalone dementia screening tools). A score of 4 or above suggests possible delirium. The cut-off of 3/4 was not derived; instead it was pre-specified in the design of the instrument. A score of 4 or more can be generated by the positive level of alertness *or* change items, *or* untestability on both cognitive items. Combinations of positive features may generate higher scores (for example, a drowsy, untestable patient who has a clear change in mental status would have a score of 12). Scores under 4 but above 0 suggest cognitive impairment. The 4AT (version 1) as used in this study is provided in Supplementary data available in *Age and Ageing* online, the Appendix, and is also available at www.the4AT.com, along with the most recent version of the guidance notes. The Italian version of the 4AT was a direct translation from the English; this was readily achievable because of the unambiguousness of the items.

### Multi-dimensional assessment

On admission, one senior geriatrician at each centre (P.M. in Monza, R.T. in Cremona) assessed each eligible patient with the 4AT. No specific training in the use of the 4AT was given. Within 48 h of admission, the same assessor administered the Alzheimer's Questionnaire (AQ), a 21-item, informant-based dementia assessment [15] to the patient's families or caregivers. The score ranges from 0 to 27, with a cut-off of 4 indicating likely cognitive impairment. The informant section of the Clinical Dementia Rating (CDR) [16] scale was administered to patients who scored >5 on the AQ. In addition, a standardised multidimensional geriatric assessment was carried out by the same assessors. Comorbidity was quantified with the Charlson index [17]. The presence of likely dementia was derived using AQ and CDR scores.

#### Reference standard diagnosis of delirium

The diagnosis of delirium was made according to DSM-IV-TR criteria [1] in each centre by an expert assessor (G.B. in Monza and A.M. in Cremona). The reference standard assessment was undertaken 15–30 min after the 4AT assessment, without knowledge of the 4AT score. The reference standard diagnostic procedure included the short Confusion Assessment Method (CAM) [18] with additional assessments as detailed below. The patient interaction started with the introduction of the assessor's name and role, followed by asking the patient's name and address, and orientation to time, place and person. Fluctuation of symptoms was ascertained through informant history from nursing staff and the patient's carers. Questions used included ‘Has there been a sudden change in patient's mental state since coming into hospital?’; ‘Does the patient seem better at any period in the day compared to other times?’; ‘Has his/her level of consciousness been altered at all - for example, has he/she been drowsy or not interacting, or perhaps agitated at times?’. Attention was evaluated using several methods. First, the patient was asked to state the days of the week forward and backwards, and to count backwards from 20 to 1. Any error in each of these tasks was considered as inattention. An additional test was the SAVEAHAART vigilance task embedded in the Confusion Assessment Method- ICU (CAM-ICU) [19], where the assessor recites the sequence of letters slowly and the patient is asked to indicate when the letter ‘A’ is recited. Inattention was defined as the presence of more than two errors, as per CAM-ICU manual. During each of these tasks, the examiner observed the patient's distractibility, comprehension and the tendency to lose the thread of conversation. Level of consciousness was assessed using the RASS [11]. The assessment of disorganised thinking was performed by asking the patient a list of pre-defined questions, such as ‘Why are you in hospital?’; ‘Will a stone float on water?’; ‘Are there fish in the sea?’. Any error in each of these tasks was considered to indicate disorganised thinking. Additionally, assessors recorded sleep-wake cycle disturbances, psychomotor abnormalities (including abnormal motor behaviour), perceptual disturbances, short- and long-term memory disturbances, psychotic symptoms and depressed mood, as derived from the clinical notes and patient interview. These assessments were used in combination against the DSM-IV-TR criteria, with the objective indicators described above supplemented by the assessors' judgement regarding the subjective features.

#### Outcome measures

The primary outcome measure was the accuracy of 4AT scores in diagnosing delirium. Secondary measures included assessing 4AT accuracy in patients with and without dementia, and the performance of each item in relation to delirium diagnosis.

#### Statistical analyses

All analyses were conducted with Stata 12.1 (StataCorp, USA). Differences in characteristics of persons with and without delirium were assessed using the *t*-test or rank-sum test for continuous variables, and the χ^2^ test for proportions. Cronbach's α was calculated for the internal reliability of the 4AT. Diagnostic test accuracy was assessed using receiver operating characteristic (ROC) curves to yield sensitivity, specificity, positive and negative likelihood ratios and area under the ROC curve (AUROC), along with 95% confidence intervals.

## Results

We studied 248 patients, 142 in Monza and 106 in Cremona. Informed consent could not be obtained for five patients. Twelve patients were excluded because the time interval between the 4AT and the reference standard assessments exceeded 30 min. The final number of patients in the study sample was 234.

Table 1 shows the clinical and demographic features. The mean age was 83.9 ± 6.1; 150 (64%) patients were women. Twenty-nine patients (12%) had delirium according to the reference standard. Dementia prevalence according to the AQ and CDR assessment was 31.2% (*n* = 74). The prevalence of delirium superimposed on dementia was 23% (*n* = 17). Participants with delirium were significantly more likely to have dementia. More patients had delirium in the OGU (25/130) than in the DRAC (7/106).

**Table 1.** Clinical characteristics of the study population, by delirium status

	No delirium		Delirium		*P-*value
*n*	207		29		
Age (mean, SD)	83.6	5.9	85.5	7.3	0.12
Sex (F, %)	131	63%	19	66%	0.85
Dementia (*n*, %)	57	28%	17	59%	<0.01
Clinical dementia rating score					
0	127	61%	10	34%	0.003
0.5	23	11%	2	7%	
1	30	14%	6	21%	
2	18	9%	5	17%	
3	9	4%	6	21%	
Charlson index (median, IQR)	2	(1–4)	2	(1–3)	0.42

**Figure 1.**
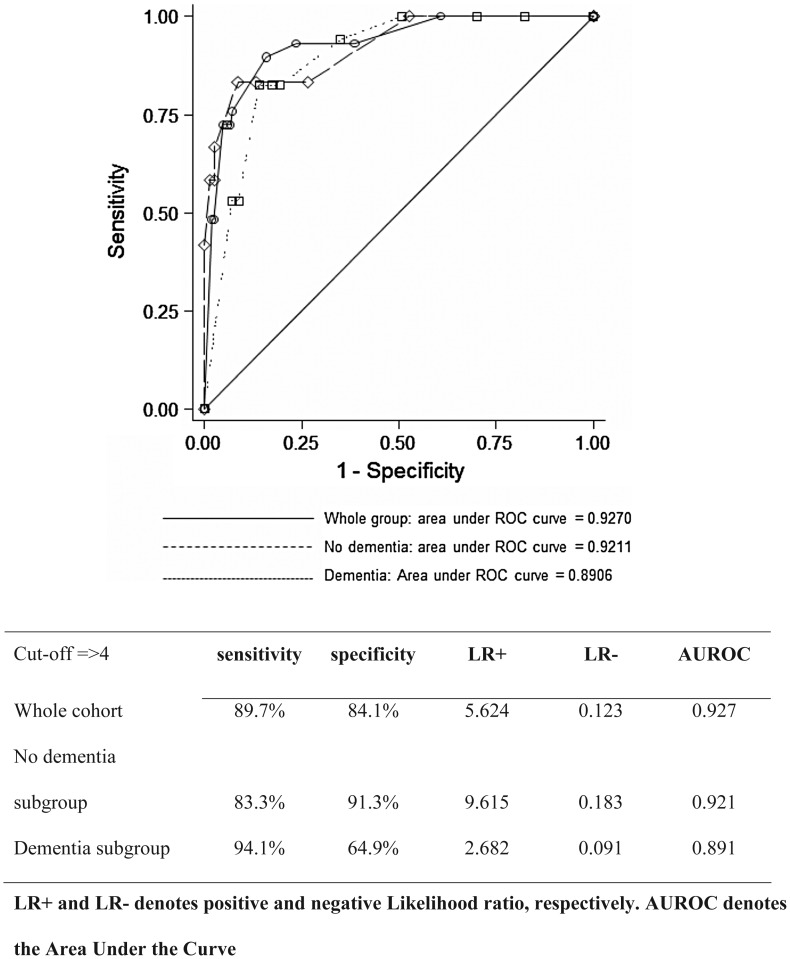
ROC comparison curve for the 4AT versus the diagnosis of delirium (DSM IV-TR criteria) in the whole population and in subgroups of patients with and without dementia.

The cut-off of 3/4 for delirium assessment, as per the 4AT specifications, was used *a priori*. In the whole sample, sensitivity was 89.7%, and specificity was 84.1%. The ROC curves are shown in Figure 1. The areas under the curves were: 0.93 in the whole population, 0.92 in patients without dementia and 0.89 in those with dementia. Other sensitivities, specificities and positive and negative likelihood ratios are given in Figure 1, showing good specificity to delirium in a dementia-free population, and good sensitivity to delirium in a dementia population. Cronbach's α for internal consistency was good, at 0.80.

Table 2 shows the diagnostic performance of each component of the 4AT. In the whole sample, the alertness and acute change/fluctuation items were highly specific (96.1 and 94.2%, respectively). The AMT4 and Months Backwards tests were sensitive to delirium (96.6 and 93.1% with a score of 1 and 89.7 and 86.2% for a score of 2, respectively); the specificity of alertness and acute change/fluctuation was excellent in persons without dementia (99.3 and 98.0%, respectively) while it was good or very good in those with dementia (87.7 and 83.9%, respectively). The sensitivity of AMT4 and Months Backwards were both high for a score of 1 (91.7 and 83.3%) and lower for a score of 2 (75.0% for both components) in persons without dementia. In those with dementia, it was highest for a score of 1 (100% for both components) and highest or very high for a score of 2 (100 and 94.1%, respectively).

**Table 2.** Diagnostic test accuracy of each component of the 4AT in relation to reference standard delirium diagnosis in the whole sample and in persons with and without dementia

	Score	Sensitivity (%)	Specificity (%)	LR+	LR−	AUROC
Whole sample						
Alertness	4	53.2	96.1	14.276	0.466	0.757
AMT4	1	96.6	54.6	2.126	0.063	0.864
	2	89.7	80.2	4.527	0.129	
Attention	1	93.1	49.8	1.853	0.139	0.850
	2	86.2	82.6	4.957	0.167	
Acute change/fluctuation	4	69.0	94.2	11.839	0.330	0.816
Dementia						
Alertness	4	50.0	99.3	75.000	0.503	0.747
AMT4	1	91.7	64.7	2.594	0.129	0.865
	2	75.0	89.3	7.031	0.280	
Attention	1	83.3	58.0	1.984	0.287	0.820
	2	75.0	89.3	7.031	0.280	
Acute change/fluctuation	1	58.3	98.0	29.167	0.425	0.782
No dementia						
Alertness	4	58.8	87.7	4.790	0.469	0.733
AMT4	1	100.0	28.1	1.390	0.000	0.781
	2	100.0	56.1	2.280	0.000	
Attention	1	100.0	28.1	1.390	0.000	0.803
	2	94.1	64.9	2.682	0.091	
Acute change/fluctuation	1	76.5	83.9	4.758	0.280	0.802

LR+ and LR−, positive and negative Likelihood ratio, respectively. AUROC, area under the curve; *n* = 234 for whole sample; *n* = 76 in dementia subgroup; *n* = 160 in 160 no dementia subgroup.

## Discussion

We found that the 4AT is a valid method of screening delirium in elderly patients on admission to geriatric wards, with overall high sensitivity and specificity. Specificity was higher in the subgroup of patients without dementia, while sensitivity was higher in those with dementia. We also found that the level of alertness and fluctuation items drove specificity to delirium, while the AMT4 (orientation) and Months Backwards (attention) items drove sensitivity to delirium.

Despite its importance, delirium continues to be misdiagnosed, detected late or missed in well over 60% of cases [9, 20]. While there are several reasons for this, one contributor is likely to be the lack of screening tools which are brief and do not rely on formal training. A recent systematic review identified 11 published screening tools [21]. However, the sensitivity of most of these tools is not satisfactory without formal training [9] and providing such training is difficult in clinical practice [22].

Acute onset and fluctuation are core diagnostic features of delirium and as expected the 4AT item assessing this feature was highly specific. Notably, the alertness item was also highly specific. A drawback of some delirium screening tools is that the scoring mechanism does not readily allow categorisation of patients whose level of arousal is too abnormal to have attention assessed by interview or cognitive testing. Yet clinical experience and the available studies [12] suggest that such patients (when not comatose) are highly likely to have a diagnosis of delirium. Therefore, in a brief screening tool, the safest approach and indeed the approach most closely aligned to the evidence is to consider such patients as having delirium unless otherwise proven otherwise [12, 23]. The 4AT provides two ways in which these patients can be described as ‘possible delirium’. First, clear abnormality of level of alertness is scored at 4. Second, if patients are considered ‘untestable’ on the AMT4 and Months Backwards tests, this also gives a score of 4. A caveat regarding this scoring mechanism is that severe chronic cognitive impairment might also yield a score of 4. However, the 4AT is a screening tool, and so ‘untestable’ patients in any case require further assessment before a definitive diagnosis can be made.

The AMT4 and Months Backwards items showed good sensitivity, but lower specificity for delirium. These findings confirm the value of cognitive tests in detecting delirium, but also show that cognitive screening alone, with these tests at least, is insufficiently specific. Nevertheless, the specificity was higher with more severe deficits, such as 2 or more errors on the AMT4, or untestability in both tests. These findings suggest that both severe disorientation and inability to perform simple cognitive tests are useful markers of delirium.

Though inattention is a core diagnostic feature of delirium, there is no consensus on how it should be assessed [23], with multiple subjective and objective methods in use clinically and in published scales. This is important because these methods vary considerably in sensitivity, reliability and other parameters, with significant implications for diagnostic thresholds. There are particular challenges where dementia is also present [24], because attentional deficits are also frequently present in dementia. The Months Backwards test was included in the 4AT as an established measure of inattention in delirium [25]. It was also included as an indicator of general cognitive impairment, because deficits in this test are also evident in dementia [14]. The present results confirm that Months Backwards is not specific to delirium, and indeed performs similarly to the AMT4 with respect to sensitivity and specificity in relation to delirium. Further work will establish if Months Backwards or other simple bedside objective tests of attention can provide sensitive and specific measures of inattention in delirium; some research suggests that this is potentially feasible [26].

A strength of this study is the provision of a clear description of exactly how the delirium diagnosis was obtained, detailing the procedure which informed scoring by DSM-IV-TR criteria. Another strength is that the study was performed outside the centre in which the 4AT was developed. Some limitations of this study must be acknowledged. The 4AT assessments were performed by experienced physicians, though no specific training in the 4AT was given. Further research is needed to assess the ease of use of 4AT among other professional groups of varying levels of seniority. We did not study the clinical outcomes in relation to ‘possible delirium’ as assessed by the 4AT. We did not assess the diagnostic accuracy of the AMT4 and Months Backwards items in relation to general cognitive impairment, though this was not the focus of this study. The prevalence of delirium in this study was comparable with that reported in previous studies (10–31% in medical inpatients and 13% in post-acute care rehabilitation [2, 4]), albeit at the lower end of the spectrum for acute inpatients. Future studies should expand the range of settings studied, including in samples with higher rates of delirium. We did not assess inter-rater reliability for the 4AT or the reference standard assessment; this should be addressed in future studies. Also, because of insufficient power, we were not able to analyse the characteristics of misclassified (false negative and false positive) patients. Finally, we did not assess the subtypes of delirium, the discriminant validity of 4AT in identifying delirium from depression, and how the scores relate to severity of delirium.

In conclusion, this study suggests that the 4AT is a valid screening tool for delirium detection in geriatrics wards. Given its brevity and practicality, it appears a useful addition to the available tests for delirium screening, in particular for use in routine clinical practice. It incorporates two items for initial testing for moderate to severe cognitive impairment which means that a separate instrument for this purpose may not be necessary. Further work is required to evaluate this possibility. Future studies in larger populations and other centres should further assess its performance, including the determination of whether detection of delirium using the 4AT may improve the clinical outcomes of patients.

**Key points**

- Although its recognition can improve patient outcomes, delirium is often undetected in acute hospitals.
- The 4 ‘A’s Test is a screening tool for delirium that is brief (generally <2 min), does not require special training, is simple to administer, and allows for the assessment of those who cannot undergo cognitive testing or interview because of severe drowsiness or agitation.
- Our study evaluated the performance of the 4AT in screening for delirium in a population of older patients, consecutively admitted over a period of 4 months to an acute geriatrics ward and a rehabilitation department. The reference standard for delirium diagnosis was the DSM-IV criteria, blind to the 4AT score.
- The results suggest that the 4AT is an effective method of screening for delirium in hospitalised older people. Its brevity and simplicity support its use in routine clinical practice. Further studies are required to confirm this.

## Supplementary data

Supplementary data mentioned in the text is available to subscribers in *Age and Ageing* online.

## Authors’ contribution

Study conception and design: Bellelli, Morandi, Davis, MacLullich. Acquisition of data: Bellelli, Morandi, Mazzola, Turco, Guerini, Torpilliesi, Del Santo, Gentile. Interpretation of results: All authors. Drafted manuscript: Bellelli, Morandi, Davis, Ryan, Cash, Annoni, Trabucchi, MacLullich. Critically revised the manuscript: All authors.

Final approval of manuscript: All authors.

## Conflicts of interest

G.B. has received honoraria from Novartis, Pfizer, Lilly and Lundbeck. D.H.J.D. is funded by the Wellcome Trust as a Research Training Fellow. M.T. has received honoraria from Novartis, Pfizer, Lilly, Nutricia and Lundbeck. A.M. has patents pending for computerised tests of inattention in delirium, and has received honoraria from Lundbeck, Shire and Novartis. The other authors report no financial conflict of interest.

The study received no specific funding. The authors' funding sources did not participate in the planning, collection, analysis or interpretation of data or in the decision to submit for publication. The investigators had full access to the data and were responsible for the study protocol, progress of the study, analysis, reporting of the study and the decision to publish.

The content is solely the responsibility of the authors.

## Supplementary Material

Supplementary Data
