# Supplementary material for: Validation of the 4AT, a new instrument for rapid delirium screening: a study in 234 hospitalised older people
Source: Age Ageing. 2014 Mar 2;43(4):496–502. doi: 10.1093/ageing/afu021 (PMC4066613; doi:10.1093/ageing/afu021)
Supplement: Supplementary Data [file supp_43_4_496__index.html]

Validation of the 4AT, a new instrument for rapid delirium screening: a study in 234 hospitalised older people — Validation of the 4AT, a new instrument for rapid delirium screening: a study in 234 hospitalised older people — Supplementary Data 

# Validation of the 4AT, a new instrument for rapid delirium screening: a study in 234 hospitalised older people

## Supplementary Data

Supplementary Data

**Files in this Data Supplement:**

- Supplementary Data - Pdf file
