# Supplementary material for: Validation of the 4AT, a new instrument for rapid delirium screening: a study in 234 hospitalised older people
Source: Age Ageing. 2014 Mar 2;43(4):496–502. doi: 10.1093/ageing/afu021 (PMC4066613; doi:10.1093/ageing/afu021)
Supplement: Supplementary Data [file supp_afu021_afu021supp.pdf]

4AT

(label)

Patient name:

Date of birth:

Patient number:

……………………………………………………………………

Date: **Time:**

Tester:

The 4A Test: screening instrument for cognitive impairment and delirium

CIRCLE

[1] ALERTNESS

This includes patients who may be markedly drowsy (eg. difficult to rouse and/or obviously sleepy during assessment) or agitated/hyperactive. Observe the patient. If asleep, attempt to wake with speech or gentle touch on shoulder. Ask the patient to state their name and address to assist rating.

Normal (fully alert, but not agitated, throughout assessment)	**0**
Mild sleepiness for <10 seconds after waking, then normal	**0**
Clearly abnormal	**4**

[2] AMT4

Age, date of birth, place (name of the hospital or building), current year.

No mistakes	**0**
1 mistake	**1**
2 or more mistakes/untestable	**2**

[3] ATTENTION

Ask the patient: "Please tell me the months of the year in backwards order, starting at December." To assist initial understanding one prompt of "what is the month before December?" is permitted.

Months of the year backwards	Achieves 7 months or more correctly	**0**
	Starts but scores < 7 months / refuses to start	**1**
	Untestable (cannot start because unwell, drowsy, inattentive)	**2**

[4] ACUTE CHANGE OR FLUCTUATING COURSE

Evidence of significant change or fluctuation in: alertness, cognition, other mental function (eg. paranoia, hallucinations) arising over the last 2 weeks and still evident in last 24hrs

No	**0**
Yes	**4**

4 or above: possible delirium +/- cognitive impairment
1-3: possible cognitive impairment
0: delirium or cognitive impairment unlikely (but delirium still possible if [4] information incomplete)

4AT SCORE [ ]

GUIDANCE NOTES Information and download: www.the4AT.com. The 4AT is a screening instrument designed for rapid and sensitive initial assessment of cognitive impairment and delirium. A score of 4 or more *suggests* delirium but is not diagnostic: more detailed assessment of mental status may be required to reach a diagnosis. A score of 1-3 suggests cognitive impairment and more detailed cognitive testing and informant history-taking are required. Items 1-3 are rated *solely on observation of the patient at the time of assessment.* Item 4 requires information from one or more source(s), eg. your own knowledge of the patient, other staff who know the patient (eg. ward nurses), GP letter, case notes, carers. The tester should take account of communication difficulties (hearing impairment, dysphasia, lack of common language) when carrying out the test and interpreting the score.

Alertness: Altered level of alertness is very likely to be delirium in general hospital settings. If the patient shows significant altered alertness during the bedside assessment, score 4 for this item. **AMT4 (Abbreviated Mental Test - 4):** This score can be extracted from items in the full AMT if done immediately before. **Acute Change or Fluctuating Course:** Fluctuation can occur without delirium in some cases of dementia, but marked fluctuation usually indicates delirium. To help elicit any hallucinations and/or paranoid thoughts ask the patient questions such as, "Are you concerned about anything going on here?"; "Do you feel frightened by anything or anyone?"; "Have you been seeing or hearing anything unusual?" In general hospital settings psychotic symptoms most often reflect delirium rather than functional psychosis (such as schizophrenia).

4AT

IL 4A Test: strumento di screening per il deficit cognitivo e il delirium

Nome del paziente:

Data di nascita:

Numero del paziente:

Data: **Ora:**

Compilatore:

Cerchiare la risposta

[1] ALLERTA

Riguarda pazienti che possono essere considerati in stato soporoso (per esempio pazienti per il quale sia difficile svegliarsi e/o che sono evidentemente soporosi durante questo test) oppure agitati/iperattivi. Osservare il paziente. Se dorme, provare a svegliarlo, parlandogli, o con un leggero tocco sulla spalla. Chiedere ai pazienti di dichiarare il proprio nome e l'indirizzo della propria abitazione per valutare il livello di collaborazione.

Normale (completamente attento, ma non agitato durante tutta la valutazione)	**0**
Moderata sonnolenza per meno di 10 secondi dopo il risveglio, poi normale	**0**
Livello di attenzione evidentemente anomalo	**4**

[2] AMT4

Età, data di nascita, luogo (nome dell'ospedale e dell'edificio), anno corrente

Nessun errore	**0**
1 errore	**1**
2 o più errori / non è possibile somministrare il test al paziente	**2**

[3] ATTENZIONE

Chiedere al paziente: "per favore, mi dica i mesi dell'anno in ordine contrario, partendo da dicembre"
Per aiutare la comprensione della domanda, è consentito inizialmente un suggerimento come: "qual è il mese prima di dicembre?"

Mesi dell'anno al contrario	nomina senza errori 7 mesi o più	**0**
	inizia, ma nomina meno di 7 mesi / si rifiuta di iniziare	**1**
	test non effettuabile (poiché il paziente è indisposto, assonnato o disattento)	**2**

[4] ACUTO CAMBIAMENTO O DECORSO FLUTTUANTE

Dimostrazione di un evidente cambiamento o di un andamento fluttuante nei seguenti domini: attenzione, comprensione o altre funzioni menta (ad esempio ossessioni e/o allucinazioni) che sono comparse nelle ultime 2 settimane e che sono ancora presenti nelle ultime 24 ore

No	**0**
Si	**4**

4 o più: possibile delirium +/- deterioramento cognitivo
1-3: possibile deterioramento cognitivo
0: improbabile **il** delirium e/o deterioramento cognitivo (ma il delirium può essere presente se il punto 4 è incompleto)

PUNTEGGIO 4

NOTE il 4AT può essere scaricato al sito: www.the4AT.com. Il 4AT è uno strumento di screening ideato per un assessment rapido del deficit cognitivo e del delirium. Uno score >4 è suggestivo di delirium ma non è diagnostico: un assessment più dettagliato dello stato mentale può essere richiesto per ottenere la diagnosi. Uno score compreso tra 1 e 3 suggerisce deficit cognitivo e richiede che vi sia una valutazione anamnestica (con un caregiver) e testistica più dettagliata. Gli items 1-3 sono valutati *solamente sulla base dell'osservazione del paziente al momento dell'assessment.* L'item 4 richiede informazioni da una o più sorgenti (ad es. la vostra conoscenza del paziente, altri membri dello staff, relazioni del medico di famiglia, caregivers, etc). L'esaminatore dovrebbe tenere in conto la presenza di deficit della comunicazione (ipoacusia, afasia, barriere linguistiche) nel portare a termine il test ed interpretare lo score. **Allerta:** un alterato stato di allerta è molto indicativo di delirium in ambito ospedaliero. Se il paziente mostra variazioni notevoli dell'allerta durante la valutazione, cerchiate lo score 4. **Cambiamento acuto o decorso fluttuante:** Una fluttuazione dello stato cognitivo può avvenire anche in assenza di delirium nei pazienti affetti da demenza, ma una fluttuazione marcata generalmente indica delirium. Per elicitare la presenza di allucinazioni o sintomi psicotici porre al paziente domande tipo "E' preoccupato/a di qualcosa in questo momento?"; "Si sente spaventato da qualcosa o qualcuno?"; "Ha sentito/visto qualcosa di strano qui?" Generalmente I sintomi psicotici in ambiente ospedaliero riflettono più frequentemente delirium che non una patologia psichiatrica funzionale (come la schizofrenia).
